# Lithium niobate photonic-crystal electro-optic modulator

**DOI:** 10.1038/s41467-020-17950-7

**Published:** 2020-08-17

**Authors:** Mingxiao Li, Jingwei Ling, Yang He, Usman A. Javid, Shixin Xue, Qiang Lin

**Affiliations:** 1 Department of Electrical and Computer Engineering, University of Rochester, Rochester, NY 14627 USA; 2 Institute of Optics, University of Rochester, Rochester, NY 14627 USA

**Keywords:** Integrated optics, Optoelectronic devices and components, Photonic crystals

## Abstract

Modern advanced photonic integrated circuits require dense integration of high-speed electro-optic functional elements on a compact chip that consumes only moderate power. Energy efficiency, operation speed, and device dimension are thus crucial metrics underlying almost all current developments of photonic signal processing units. Recently, thin-film lithium niobate (LN) emerges as a promising platform for photonic integrated circuits. Here, we make an important step towards miniaturizing functional components on this platform, reporting high-speed LN electro-optic modulators, based upon photonic crystal nanobeam resonators. The devices exhibit a significant tuning efficiency up to 1.98 GHz V^−1^, a broad modulation bandwidth of 17.5 GHz, while with a tiny electro-optic modal volume of only 0.58 *μ*m^3^. The modulators enable efficient electro-optic driving of high-Q photonic cavity modes in both adiabatic and non-adiabatic regimes, and allow us to achieve electro-optic switching at 11 Gb s^−1^ with a bit-switching energy as low as 22 fJ. The demonstration of energy efficient and high-speed electro-optic modulation at the wavelength scale paves a crucial foundation for realizing large-scale LN photonic integrated circuits that are of immense importance for broad applications in data communication, microwave photonics, and quantum photonics.

## Abstract (web summary)

Lithium niobate (LN) devices are promising for future photonic integrated circuits. Here, the authors demonstrate an electro-optic LN modulator with a very small modal volume based on photonic crystal resonator architecture.

## Introduction

High-speed electro-optic modulation underlies many important applications ranging from optical communication^1^, microwave photonics^2^, computing^3^, frequency metrology^4^ to quantum photonics^5^. A variety of approaches have been employed for electro-optic modulation, such as carrier plasma dispersion^6,7^, electro-absorption^8,9^, and Pockels effect^1,10^, the latter of which is particularly interesting since the Pockels effect offers an ultrafast and pure refractive-index modulation over an extremely broad optical spectrum while without introducing extra loss. The best-known electro-optic Pockels material is probably lithium niobate (LiNbO_3_, LN), which has been widely used in telecommunication^1^. Recently, thin-film monolithic LN^11,12^ emerges as a promising platform, where low-loss and high-quality photonic integration together with the strong Pockels effect enables superior modulation performance^13–26^, showing great potential as an excellent medium for photonic integrated circuits and future photonic interconnect.

Power efficiency is crucial for the application of electro-optic modulator (EOM), which depends sensitively on the physical size of the device^27^. Scaling an EOM down to a small footprint would reduce the device capacitance and thus decrease the switching energy^27,28^, which is indispensable for all practical applications. A variety of approaches have been explored for this purpose, including plasmonic structures^29,30^, slotted dielectric waveguides^10^, photonic micro-/nanoresonators^6,31^, etc. Among various device geometries, photonic-crystal nanoresonators are particularly beneficial in this regard, given their exceptional capability of controlling light confinement and light–matter interactions on the sub-wavelength scale. In the past decade, photonic-crystal EOMs have been developed on various material platforms such as silicon^32–34^, GaAs^35^, InP^36^, polymers^37,38^, ITO^39^, etc. For LN, however, the EOMs developed so far^1,13–26^ generally exhibit significant dimensions, leading to significant power required to drive the EOMs. Although attempts have been made to explore the electro-optic effect in LN photonic crystals^40–42^, the low device quality and poor optoelectronic integration unfortunately limit seriously the operation speed. To date, it remains an open challenge in realizing a high-speed and energy-efficient modulator at the wavelength scale on the monolithic LN platform.

Here we report high-speed and energy-efficient LN photonic-crystal EOMs, which exhibits a tiny electro-optic modal volume of only  ~0.58 μm^3^, the smallest among all high-speed LN EOMs ever reported^1,13–26^, to the best of our knowledge. The sub-wavelength-scale EOM cavity enables compact optoelectronic integration to achieve not only a high electro-optic tuning efficiency up to 16.0 pm V^−1^ (corresponding to 1.98 GHz V^−1^) that is significantly beyond other LN EOM resonators^13–16,18,19,23,26^, but also a large modulation bandwidth up to 17.5 GHz that reaches the photon-lifetime limit of the EOM cavity. The fully on-chip design achieves a full-swing extinction ratio of 11.5 dB. With these devices, we are able to realize efficient driving of the optical mode in both adiabatic sideband-unresolved and non-adiabatic sideband-resolved regimes, and to observe the transition in between. As an example application, we demonstrate electro-optic switching of non-return-to-zero (NRZ) signal at a rate of 11 Gb s^−1^, with a switching energy as low as 22 fJ per bit that is more than one order of magnitude smaller than other LN EOMs^1,13–26^.

## Results

### Device modeling

Recently, there have been significant advance in high-*Q* LN photonic-crystal nanoresonators^43–46^, which led to the demonstration of intriguing phenomena and functionalities such as photorefraction quenching^43^, harmonic generation^44^, piezo-optomechanics^45^, and all-optical resonance tuning^46^.

**Fig. 1:**
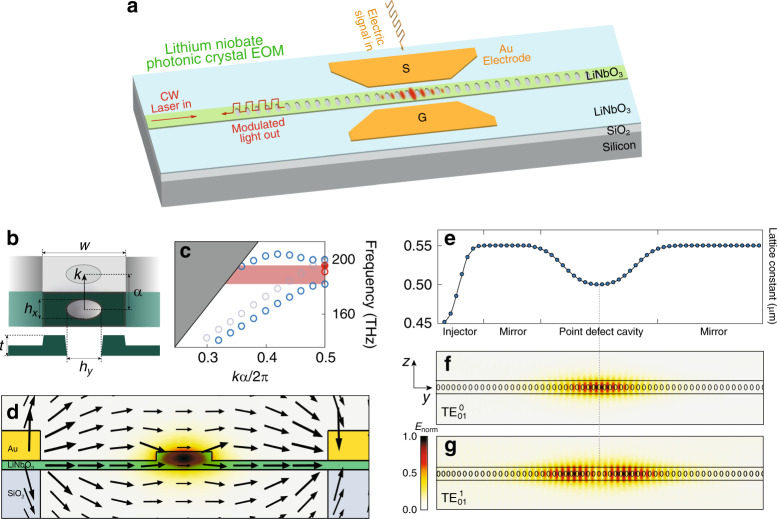
Design of LN photonic-crystal EOM. **a** Schematic of the LN photonic-crystal EOM. **b** The structure of the unit cell (top: top view; bottom: cross-sectional view). The LN photonic-crystal nanobeam has a width of *w* = 1200 nm, layer thickness of *t* = 300 nm, and a partially etched wing layer with a thickness of 150 nm. The elliptical hole has dimensions of *h*_*x*_ =  270 nm and *h*_*y*_ = 490 nm, and a fully etched depth of 300 nm. *a* is the lattice constant. The full cross-section is shown in **d**. **c** Dispersion property of the partially etched LN photonic-crystal nanobeam, simulated by the finite element method (FEM). The blue open circles show the dielectric and air bands. The red solid and open circles denote the fundamental and second-order TE-like cavity modes shown in **f** and **g**. Our simulations show that there exhibits another mode with eigenfrequency within the band gap (gray open circles). This mode, however, has only negligible perturbation to the dielectric mode due to distinctive spatial symmetry, thus not affecting the quality of the defect cavity mode. **d** Cross-sectional schematic of the EOM structure, where the arrow profile shows the radio frequency (RF) electric field distribution and the color profile shows the optical cavity mode field distribution, both simulated by the FEM method. **e** Lattice constant *a* as a function of position, which is optimized for low insertion loss together with high radiation-limited optical *Q*. **f** Top view of the FEM-simulated optical mode field profile of the fundamental TE-like cavity mode ${\mathrm{{TE}}}_{01}^{0}$. The left inset shows the orientation of the LN crystal where the optical axis is along the *z* direction. **g** Simulated optical mode field profile of the second-order TE-like cavity mode ${\mathrm{{TE}}}_{01}^{1}$. The scale bar on the left represents the strength of normalized electrical field (*E*_norm_) for **d**, **f**, **g**.

For EOM, we adopt one-dimensional photonic-crystal nanobeam as the basic underlying structure (Fig. 1a) since it supports compact optical and electrical integration to enhance the electro-optic response. Due to the high permittivity of LN at radio frequency, the commonly used full surrounding air cladding^43,45,46^ is not suitable for EOM since it would significantly reduce the coupling between the optical and electric fields. To maximize the electro-optic interaction, we utilize a partially etched structure with a rib-waveguide-like cross-section, leaving a 150-nm-thick wing layer for the electrodes to sit on (Fig. 1a, d). Although the breaking of the mirror symmetry along the normal direction of the device plane considerably alters the band gap of the photonic crystal (Fig. 1c), optimization of the photonic potential via an appropriate pattern of lattice constant (Fig. 1e) is still able to produce a well-confined point-defect cavity, with a simulated optical *Q* of  ~10^5^ for the fundamental transverse-electric-like (TE-like) cavity mode, ${\mathrm{{TE}}}_{01}^{0}$, shown in Fig. 1f. The cavity mode exhibits an extremely small electro-optic modal volume of 1.52(*λ*/*n*)^3^ ~0.58 μm^3^ (where *n* is the refractive index of LN).

The photonic-crystal cavity is oriented along the *y*-axis such that the dominant optical field is in parallel with the optical axis of underlying LN medium (Fig. 1f), so as to take the advantage of the largest electro-optic component *r*_33_ of LN. The electrodes are designed to be placed close to the photonic-crystal resonator (Fig. 1d) to maximize the in-plane electric field *E*_*z*_, while preventing potential loss induced by metal absorption, which results in a significant electro-optic tuning efficiency of 1.81 GHz V^−1^, simulated by the finite element method (see “Methods” for simulation details). The electrodes are designed to have a length of 30 μm to ensure a full coverage of the applied electric field over the entire photonic-crystal structure. Numerical simulations show that the device exhibits a small capacitance *C* of *C* = ~22 fF, which is more than one order of magnitude smaller than other LN EOMs^1,13–26^. Therefore, we expect our devices to have much higher energy efficiency, as will be shown in the following sections.

For simplicity of testing, the EOM is designed such that light is coupled into and out of the EOM via only one side of the cavity (Fig. 1a). As such, the photonic-crystal mirror on the right side of the defect cavity is designed to be of 100% reflection, while that on the left side has decreased number of holes (Fig. 1e) to enable a partial reflection/transmission, with the hole number optimized for a critical coupling to the cavity. To support on-chip integration, light is coupled to the EOM cavity via an on-chip waveguide (Fig. 1a), where an injector section (Fig. 1e), with the lattice constant varying from 450 to 550 nm, is designed and placed in front of the left mirror to reduce the coupling loss.

**Fig. 2:**
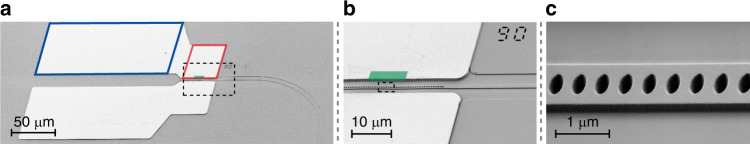
Scanning electron microscopic (SEM) image of a fabricated EOM device. **a** Full SEM image of the whole-device structure. The region highlighted in red is the electrode used to drive the photonic-crystal nanoresonator. That highlighted in blue indicates the large metal pad used for contacting the RF probe. The green region indicates the electrode that can be shrunk to in the future design. **b** Zoom-in image of the photonic-crystal resonator and electrodes, corresponding to the dashed rectangular region in **a**. **c** Further zoom-in image showing the detailed structure of the photonic-crystal defects cavity, corresponding to the dashed rectangular region in **b**.

**Fig. 3:**
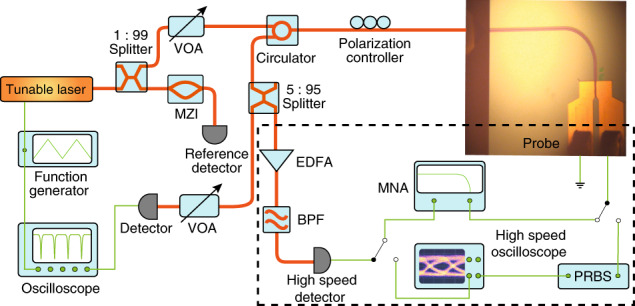
Experimental testing setup. Light is coupled into and out of the EOM chip via one lensed fiber. The inset shows an optical microscopic image of an EOM with the RF probe in contact. The equipment in the highlighted dashed box is used for characterizing the performance of electro-optic modulation. VOA variable optical attenuator, MZI Mach–Zehnder interferometer, EDFA erbium-doped fiber amplifier, BPF bandpass filter, MNA, microwave network analyzer, PRBS pseudo-random binary sequence source.

Figure 2 shows a fabricated device (see “Methods” for the details of device fabrication). The large metal pads (highlighted in blue box) are used simply as the contacts for the air-coplanar probe (Formfactor Acp65-A-GSG-100) for applying the RF driving signal (see also the inset of Fig. 3). The impedance of the metallic structure is optimized to minimize the coupling loss of the RF signal from the pads to the device. The high quality of device fabrication as indicated by the device images implies high performance of the EOM, as we will show below.

### Device characterization and electro-optic properties

To characterize the optical and electro-optic properties of the devices, a continuous-wave tunable laser (Santec TSL-510) was launched onto the chip via a lensed fiber. The light reflected from the EOM was collected by the same lensed fiber, routed by a circulator, and then delivered to a photodiode for detection. Figure 3 illustrates the schematic of the experimental testing setup, where the inset shows an optical image of the device with the RF probe in contact. The insertion loss from the on-chip coupling waveguide to the photonic-crystal cavity is measured to be around 2.2 dB, calibrated by subtracting the facet coupling and circulator transmission loss. To characterize the performance of high-speed modulation, the majority of the modulated light output was amplified by an erbium-doped fiber amplifier to boost the power, passed through a bandpass filter to remove the amplifier noise, and was then detected by a high-speed detector (New Focus 1024). The detector output was recorded either by a microwave network analyzer (Keysight N5235B) for characterizing the modulation bandwidth or by a sampling oscilloscope module (Keysight 54754A) to record the eye diagram of the switching signal.

**Fig. 4:**
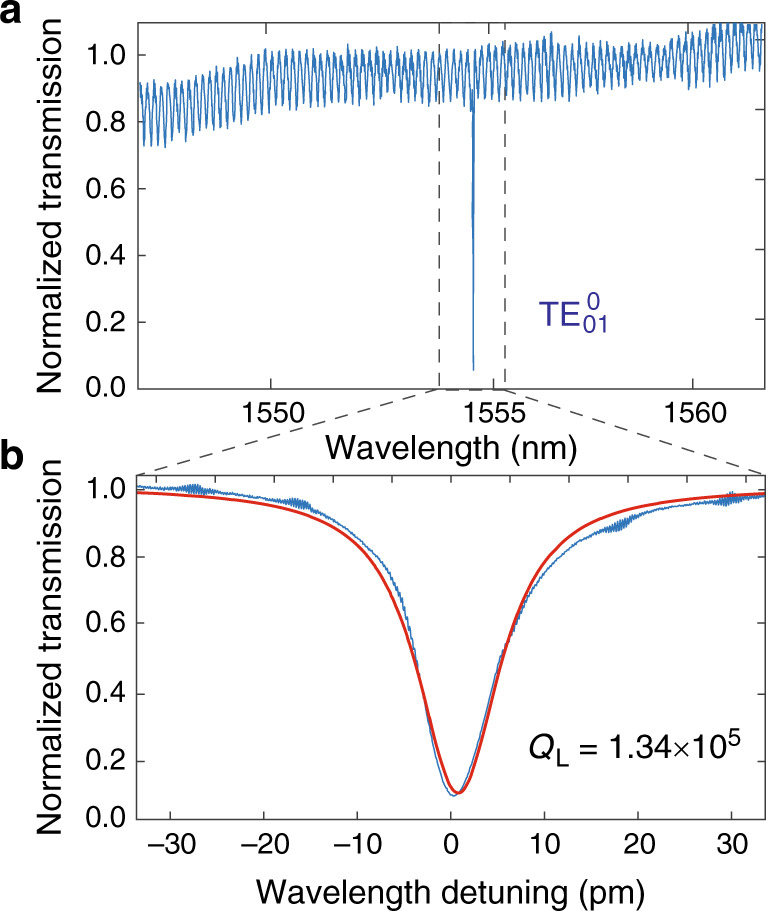
Linear optical property of a fabricated LN photonic-crystal EOM. **a** Laser-scanned transmission spectrum in the telecom band. **b** Detailed transmission spectrum of the fundamental TE-like cavity mode ${\mathrm{{TE}}}_{01}^{0}$ at a wavelength of 1554.47 nm, with the experimental data shown in blue and the theoretical fitting shown in red.

Figure 4a shows the transmission spectrum of an EOM when the laser is scanned in the telecom band. The device exhibits a resonance at 1554.47 nm, which corresponds to the fundamental TE-like cavity mode ${\mathrm{{TE}}}_{01}^{0}$ (Fig. 1f). As shown in Fig. 4b, the ${\mathrm{{TE}}}_{01}^{0}$ mode exhibits a high loaded optical *Q* (*Q*_L_) of 1.34 × 10^5^, which is very close to our numerical simulation, indicating the negligible impact of the electrodes on the optical quality. The cavity resonance exhibits a coupling depth of 93%, corresponding to a full-swing extinction ratio of 11.5 dB. This value can be improved in the future by further optimizing the partially reflective photonic-crystal mirror (Fig. 1e). The device also exhibits a second-order TE-like cavity mode ${\mathrm{{TE}}}_{01}^{1}$ (Fig. 1g) at 1604.13 nm (not shown) with a loaded optical *Q* of 3.03 × 10^4^.

**Fig. 5:**
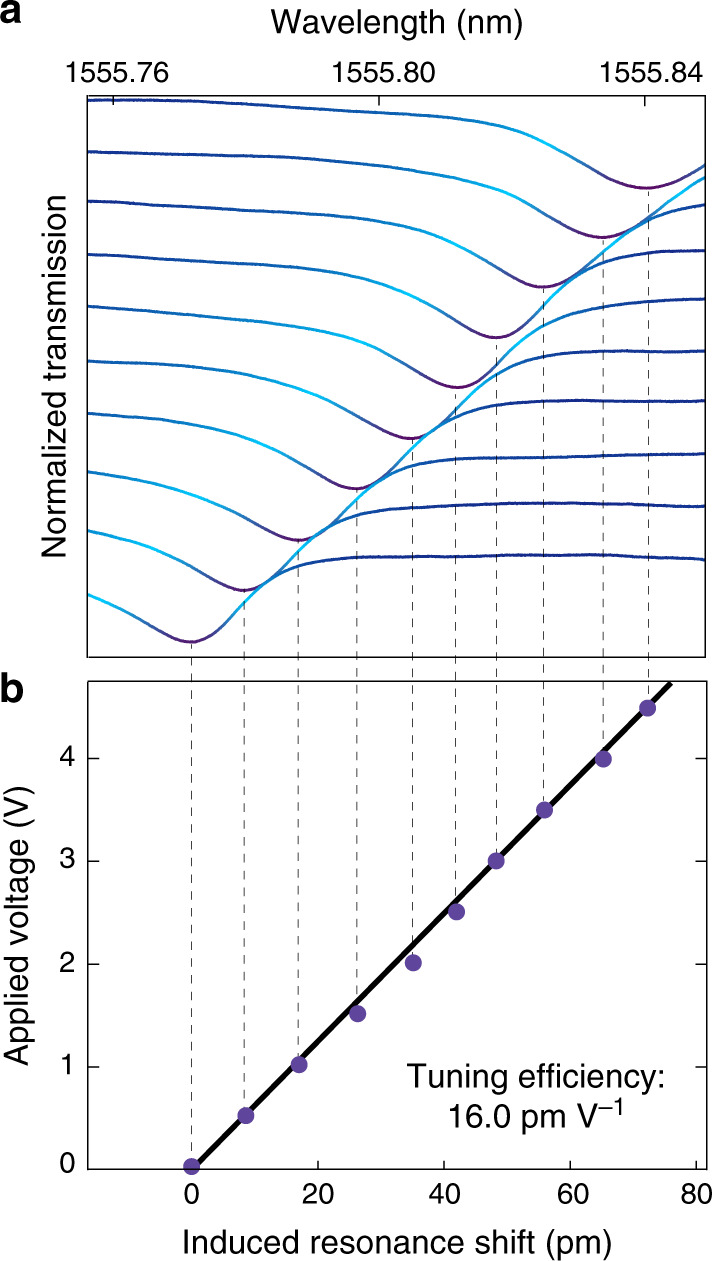
Electro-optic tuning property of an LN photonic-crystal EOM. **a** Recorded transmission spectrum of the EOM cavity as a function of applied DC voltage from 0 to 4.5 V, with a voltage step of 0.5 V. **b** Recorded resonance shift as a function of applied DC voltage, where the experimental data are shown in black dots and the blue line is a linear fitting to the data.

To show the electro-optic tuning property, we applied a DC voltage to the chip and monitored the cavity transmission spectrum of the ${\mathrm{{TE}}}_{01}^{0}$ mode. As shown in Fig. 5a, the cavity resonance tunes smoothly with the applied voltage, without any degradation to the lineshape or coupling depth, clearly showing the pure dispersive electro-optic tuning as expected from the Pockels effect. We have applied a voltage of 25 V to the device (not shown in the figure) and did not observe any degradation. Figure 5b shows a clear linear dependence of the induced resonance wavelength shift on the applied voltage, from which we obtained a tuning slope of 16.0 pm V^−1^ (corresponding to a frequency tuning slope of 1.98 GHz V^−1^), close to our design. This value is significantly larger than those in other LN EOM resonators^13–16,18,19,23,26^, which is primarily benefited from the strong optical field confinement, large optical and electric field overlap, and the resulting compact optical and electric integration offered by our devices. The pure linear electro-optic tuning shown in Fig. 5 implies that the linearity of electro-optic modulation in the devices would reach the intrinsic limit determined by the fundamental Lorentzian shape of the cavity resonance^47^.

### Electro-optic modulation

The high efficiency of electro-optic tuning together with the high optical quality of the EOM resonator enables efficient electrical driving of the optical mode into different dynamic regimes. To show this phenomenon, we applied a sinusoidal RF signal at a certain frequency to the EOM and monitored the transmission spectrum of the device by scanning laser back and forth across the cavity resonance. The laser wavelength is scanned at a repetition rate of  ~15 Hz, so we primarily monitored the time-averaged cavity transmission.

**Fig. 6:**
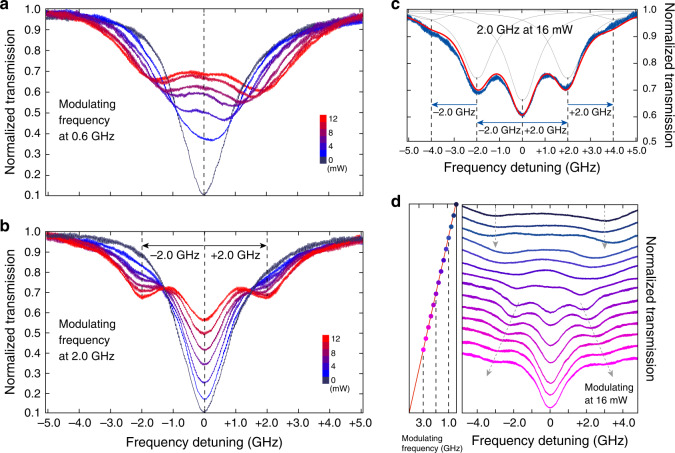
Electro-optic modulation of a high-*Q* optical cavity resonance. **a** Recorded transmission spectra of the ${\mathrm{{TE}}}_{01}^{0}$ cavity mode with RF driving signal at seven different powers from 0 to 12 mW, with a power step of 2 mW, modulated at 0.6 GHz. **b** Same as **a** but with a modulation frequency of 2.0 GHz. **c** Detailed spectrum (blue) with RF driving signal at 2.0 GHz with a power of 16 mW. The gray curves show the created individual sidebands with Lorentzian-shape resonances and the dashed vertical lines indicates their relative frequency positions. The sum of all gray curves is showed in red, which is fitted by the theory. **d** Recorded transmission spectra at different RF modulation frequencies varying from 0.4 to 3.0 GHz, with a frequency step of 0.2 GHz. The RF driving power is 16 mW.

When the EOM is driven at a modulation frequency of 600 MHz much smaller than the cavity linewidth of 1.4 GHz, increasing the driving power simply broadens the transmission spectrum into one with two shallow side lobes, as shown in Fig. 6a, with a broadened spectral linewidth dependent on the driving power. This is a typical signature of resonance modulation in the sideband-unresolved regime, where the cavity resonance follows adiabatically the electric driving signal in a sinusoidal fashion, resulting in a broadened average transmission spectrum (Fig. 6a).

When the modulation frequency is increased to 2.0 GHz greater than the cavity linewidth, the cavity is too slow to follow the electro-optic modulation, which results in the frequency conversion of photons into sidebands with frequency separation equal to the modulation frequency. Consequently, the transmission spectrum transforms into a multi-resonance spectrum (Fig. 6b). Increasing the electrical driving power now does not perturb the positions of the resonance dips, but rather changes their relative magnitudes since the magnitudes of the created sidebands depends on the driving amplitude^48^. This phenomenon is shown more clearly in Fig. 6c, where a driving power of 16 mW (corresponding peak-to-peak driving voltage, *V*_pp_, of *V*_pp_ = 2.5 V) splits the cavity resonance into five with notable magnitudes (black curve), resulting in a cavity transmission with five side lobes (blue curve).

Electro-optic modulation enables arbitrary modulation of cavity resonance within the bandwidth allowed by the driving circuit. This is in strong contrast to piezoelectric acoustic modulation which is confined to the vicinity of mechanical resonance frequency^45,49,50^. Such flexibility allows us to observe direct transition between the adiabatic driving regime and the non-adiabatic regime simply by continuously sweeping the modulation frequency to across the cavity linewidth. Figure 6d shows an example. When the modulation frequency is below 1.0 GHz, The transmission spectrum remains fairly similar regardless of modulation frequency, as expected from the adiabatic driving discussed above. However, when the modulation frequency is tuned above 1.0 GHz towards the cavity linewidth, the two side lobes moves towards each other and the spectral shape is considerably distorted, until around 1.8 GHz where the transmission spectrum splits into three lobes, with the two side lobes located about 1.8 GHz from the center. Further increase of the modulation frequency shifts apart the two side lobes accordingly, with amplitude decreased, while the position of the center lobe remains unchanged, as expected from the non-adiabatic driving. The flexible electro-optic modulation shown here may offer a convenient method for controlling the spectrotemporal properties of photons inside the cavity and for creating exotic quantum states^48^ that are crucial for quantum photonic applications.

### Electro-optic switching

**Fig. 7:**
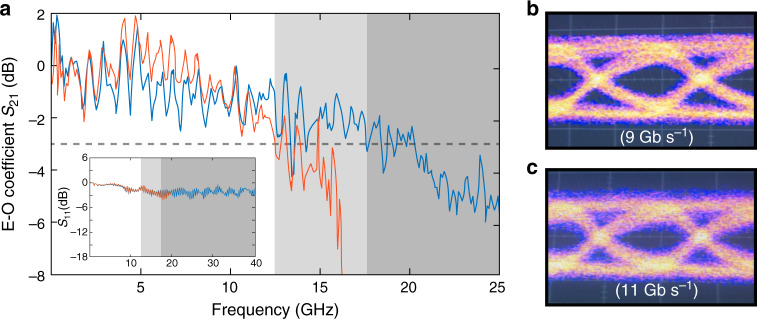
High-speed electro-optic switching. **a** Recorded scattering parameter *S*_21_ for two devices with optical *Q* of  ~14,000 (blue curve) and  ~20,000 (orange curve), respectively. The gray regions represents the 3-dB bandwidth limit for two devices, respectively, and the dashed line indicates the 3-dB limit of *S*_21_. The inset shows the *S*_11_ reflection scattering parameter for both devices. **b**, **c** Eye diagrams of the photonic-crystal EOM output, measured with 2^7^−1 NRZ PRBS with a driving voltage of *V*_pp_ = 2 V. The laser wavelength was locked at half wave into the cavity resonance.

The electro-optic modulation demonstrated in the previous section indicates the potential high-speed operation of the EOMs. To show this feature, we selected another similar device on the same chip, which has a lower loaded optical *Q* of 14,000. Figure 7a shows the electro-optic modulation response of the device (blue curve), which exhibits a 3-dB modulation bandwidth up to around 17.5 GHz. This value primarily reaches the photon-lifetime limit of the EOM cavity (~11 ps), as the electrode circuit has much broader spectral response as indicated by the flat *S*_11_ reflection spectrum shown in the inset of Fig. 7a. As the modulation bandwidth is primarily related to the optical *Q* of the device, it can be engineered flexibly for different application purposes, simply by choosing device with appropriate optical *Q*. The orange curve in Fig. 7a shows another example of a device with optical *Q* of 20,000, which exhibits a 3-dB bandwidth of about 12.5 GHz.

The broad modulation bandwidth of these devices would thus enable high-speed electro-optic switching. As an example application, we applied NRZ signal with a (2^7^−1)-bit pseudo-random binary sequence (PRBS) to an EOM with a *V*_pp_ of 2.0 V. Figure 7b, c shows the recorded eye diagrams at two different bit rates of 9 and 11 Gb s^−1^, respectively, which show clear open eyes. The demonstrated bit rate is currently limited by our PRBS generator (Agilent 70843B) which has a maximum bit rate of 12 Gb s^−1^. However, negligible degradation observed between Fig. 7b, c implies that the EOM could operate at higher bit rates, which will left for future demonstration. The bit-switching energy for NRZ signal is given by $\frac{1}{4}C{V}_{{\rm{pp}}}^{2}$ (ref. ^27^), which is about 22 fJ per bit in our EOM. This value is the smallest switching energy ever reported for LN EOMs^1,13–26^, clearly showing the high energy efficiency of our devices.

## Discussion

The energy efficiency of the LN photonic-crystal EOM can be further improved since our current devices are not optimized. For example, the capacitance of our device can be significantly decreased since the majority of the metallic parts in the current devices are used for coupling the RF driving signal, which can be removed in a future on-chip integration design. The 50-μm width of the electrode (Fig. 2, red box) is used primarily for impedance matching to the large metal pad for probe contact, which can be decreased to 3 μm for a fully on-chip operation^36^. On the other hand, the 30-μm length of the electrode is overly conservative since it covers the full length of photonic-crystal structure including the injector, mirrors, and the cavity (Figs. 1e and  2). Essentially, only the 10-μm long point-defect cavity requires electric driving to achieve electro-optic modulation. Therefore, the electrodes can be shrunk to 10 × 3 μm^2^, which would reduce the capacitance considerably to  ~0.27 fF (~ 1.0 fF if including the integrated wires^36^), according to our FEM simulations. On the other hand, the electrodes are currently placed far from the photonic-crystal cavity so as to leave the optical mode intact to achieve a high optical *Q*. For the application of high-speed electro-optic switching, our simulations show that the electrode-waveguide spacing can be decreased to 1.5 μm for an optical *Q* of  ~5000 (corresponding to a modulation bandwidth of  ~45 GHz), which will improve the modulation efficiency to 2.38 GHz V^−1^ (simulation details in “Methods”). We expect that these optimization would significantly improve the energy efficiency of the LN photonic-crystal EOM, further decreasing the switching energy down to sub-femtoJoule level.

In the current EOMs shown above, light is coupled into and out of the EOMs via a same side of the cavity, which is not convenient in practice since a circulator is required to separate the modulated light for the laser input. This can be changed simply by engineering the photonic-crystal mirror on the other side to function as the output port. On the other hand, the full-swing extinction ratio of the EOMs is primarily determined by the external coupling of light to the modulator cavity. The extinction ratio can be significantly improved by further optimization of the photonic-crystal mirrors (Fig. 1e) to achieve a critical coupling.

In summary, we have demonstrated high-speed LN EOMs with a broad modulation bandwidth of 17.5 GHz, a significant tuning efficiency up to 1.98 GHz V^−1^, and an electro-optic modal volume as small as 0.58 μm^3^. We believe this is the first LN EOM ever reported with such combined device characteristics and modulation performance. With these devices, we are able to demonstrate efficient electrical driving of high-*Q* cavity mode in both adiabatic and non-adiabatic regimes and to observe transition in between. We are also able to achieve high-speed electro-optic switching of at 11 Gb s^−1^, with switching energy as low as 22 fJ per bit. The demonstration of energy efficient and high-speed EOM at the wavelength scale paves an important step for device miniaturization and high-density photonic integration on the monolithic LN platform, which is expected to find broad applications in communication, computing, microwave signal processing, and quantum photonic information processing.

## Methods

### Device fabrication

The devices were fabricated on a 300-nm-thick x-cut single-crystalline LN thin film bonded on a 3-μm silicon dioxide layer sitting on a silicon substrate (from NanoLN). The photonic-crystal hole structure was patterned with ZEP-520A positive resist via electron-beam lithography, which was then transferred to the LN layer with an Ar^+^ plasma milling process to etch down the full 300-nm depth. The resist residue was removed by a further O^+^ plasma etching. A second exposure is then performed to define the waveguide structure, which is partially etched by 150 nm with the same process. After the residue removal, we used diluted hydrofluoric acid to undercut the buried oxide layer to form a suspended photonic-crystal membrane structure (Fig. 1d). The metal electrode layer (10 nm Ti/500 nm Au) was deposited by an electron-beam evaporator and the electrode structure was formed by a lift-off process via ZEP-520A.

### Design and modeling of electro-optic coupling

**Fig. 8:**
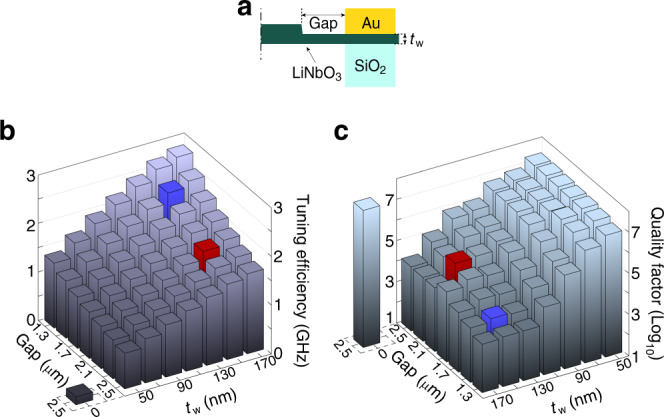
Numerical modeling of electro-optic coupling. **a** Schematic of half of the cross-section of the EOM structure. “Gap” denotes the spacing between the gold electrode and the LN cavity, and “*t*_w_” denotes the thickness of the waveguide wing layer. **b, c** Electro-optic tuning efficiency and optical quality factor of the device as a function of Gap and *t*_w_, simulated by the FEM method. The red column represents the parameter design used for the EOMs demonstrated in the main text. The blue column shows another design with broader bandwidth and enhanced electro-optic coupling. The individual column at the left of each plot indicates the case when *t*_w_ = 0 μm and gap = 2.5 μm, for a device with full surrounding air cladding.

One-dimensional photonic-crystal nanobeams exhibit exceptional capability of controlling light confinement. A typical design requires a full air cladding to improve the optical quality factor^43,45,46^. However, the dielectric constant of LN is  ~28 at a microwave frequency, much larger than that of air. As a result, a full air cladding would strongly limits the electro-optic coupling, leading to a low efficiency of electro-optic tuning as indicated by the individual black column in Fig. 8b. To improve the electro-optic coupling, we utilize a partially etched structure with a rib-waveguide-like cross-section (Figs. 1d and 8a). Figure 8b, c shows the numerically simulated tuning efficiency and the corresponding optical *Q*, respectively.

As shown in Fig. 8b, increasing the thickness, *t*_w_, of the wing layer will improve the electro-optic tuning since it enhances the amplitude of the driving electric field inside the LN photonic-crystal cavity. It, however, degrades considerably the optical *Q* of the cavity (Fig. 8c) due to the decrease of optical mode confinement. On the other hand, placing the electrode closer to the photonic-crystal cavity will strengthen as well the driving electric field inside the cavity and thus enhance the electro-optic tuning (Fig. 8b), which, however, might sacrifice the optical *Q* due to metallic losses. Thanks to the strong light confinement, we are able to place the electrode fairly close to the cavity without introducing extra optical loss (Fig. 8c). A notable decrease of optical *Q* is not observed until the gap is reduced to below 2.3 μm. With a balance between the optical *Q* and the electro-optic tuning efficiency, we adopt a design with a 2.1-μm gap and a 150-nm-thick wing layer to achieve the performance demonstrated in this paper, which is highlighted in red in Fig. 8b, c. However, Fig. 8 shows that there are considerable rooms to further improve the EOM performance. As an example, a gap of  ~1.5 μm would not only result in an optical *Q* of  ~5000 that helps increase the operation bandwidth to  ~45 GHz, but also improve the electro-optic tuning efficiency by about 31% to 2.38 GHz V^−1^, as highlighted in blue in Fig. 8b, c.

## Data Availability

The data that support the findings of this study are available from the corresponding author upon reasonable request.
